# Cardiac incoordination induced by left bundle branch block: its relation with left ventricular systolic function in patients with and without cardiomyopathy

**DOI:** 10.1186/1476-7120-6-39

**Published:** 2008-08-05

**Authors:** Miguel Quintana, Samir Saha, Satish Govind, Lars Åke Brodin, Francesca del Furia, Vicente Bertomeu

**Affiliations:** 1 Institution of Laboratory Medicine, Department of Cardiology, Hospital de Torrevieja, Spain; 2 Karolinska University Hospital, Huddinge, Stockholm, Sweden; 3 Departamento de Cardiología, Hospital Universitario de San Juan, Alicante, Spain

## Abstract

**Background:**

Although left bundle branch block (LBBB) alters the electrical activation of the heart, it is unknown how it might change the process of myocardial coordination (MC) and how it may affect the left ventricular (LV) systolic function. The present study assessed the effects of LBBB on MC in patients with LBBB with and without dilated (DCMP) or ischemic cardiomyopathy (ICMP).

**Methods:**

Tissue Doppler echocardiography (TDE) was performed in 86 individuals: 21 with isolated LBBB, 26 patients with DCMP + LBBB, 19 patients with ICMP + LBBB and in 20 healthy individuals (Controls). MC was assessed analyzing the myocardial velocity profiles obtained from six basal segments of the LV using TDE. The LV systolic function was assessed by standard two-dimensional echocardiography and by TDE.

**Results:**

Severe alterations in MC were observed in subjects with LBBB as compared with controls (P < 0.01 for all comparisons); these derangements were even worse in patients with DCMP and ICMP (P < 0.001 for comparisons with Controls and P < 0.01 for comparison with individuals with isolated LBBB). Some parameters of MC differed significantly between DCMP and ICMP (P < 0.01). A good or very good correlation coefficient was found between variables of MC and variables of LV systolic function.

**Conclusion:**

LBBB induces severe derangement in the process of MC that are more pronounced in patients with cardiomyopathies and that significantly correlates with the LV systolic function. The assessment of MC may help in the evaluation of the etiology of dilated cardiomyopathy.

## Introduction

Left bundle branch block (LBBB) alters the pattern of electrical activation of the heart [1-4] and disturbs the left ventricular (LV) systolic function[1,5-7] even in absence of other cardiovascular diseases[8]. The presence of LBBB in patients with dilated (DCMP) or ischemic cardiomyopathy (ICMP) implies a progressive worsening of the LV systolic function and prognosis [9-13]. In these patients cardiac resynchronization therapy (CRT) improves short- and long-term hemodynamics, functional capacity, quality of life and survival [14-18]. However, even following simple electrocardiographic and echocardiographic selection criteria for CRT[19,20] one third to one fourth of the patients do not respond to or even worsens after CRT[17,21-23]. Consequently, different techniques, including Tissue Doppler echocardiography (TDE) have been used to detect inter and intra-ventricular cardiac dyssynchrony, to evaluate its effects on LV systolic function, and to assess the effects of CRT [24-28]. Although a delayed mechanical contraction of some LV walls plays an important role in the LV hemodynamics, it is less known how a delayed electrical activation might affect the process of myocardial coordination (MC), defined as the synchronicity of time-related events occurring before mechanical contraction and ventricular filling, and how an alteration in MC might affect the LV contraction and hemodynamics. The present study was thus designed to assess the physiological basis of MC in patients with LBBB with or without DCMP or ICMP by means of TDE.

## Patients and methods

### Patients and controls

The studied population consisted of 86 individuals: 21 with isolated LBBB but otherwise healthy, 26 patients with DCMP and LBBB, 19 patients with ICMP and LBBB, and 20 healthy individuals (Controls). The controls, being part of a study in the geriatric population[29], were asymptomatic without treatment with cardiovascular pharmacological agents and had a normal rest ECG, a normal standard echocardiogram, and a normal exercise test. Individuals with LBBB were also asymptomatic and were recruited from an ECG-database; none of them were on treatment with cardiovascular pharmacological agents and all of them had been referred to a rest ECG as a routine procedure before a non-cardiovascular surgical intervention. Among patients with DCMP and ICMP, 84% were on treatment with diuretics, 78% were on beta-blocker agents, 72% were on angiotensine converting enzyme inhibitors or angiotensine II receptor blockers, 67% were on aspirin, 42% on digoxin, and 34% on oral anticoagulants. All participants gave a written consent and the ethical committee at the Karolinska University Hospital, Huddinge, approved the study.

### Standard echocardiography

Echocardiography was performed with a System V™ equipment (General Electric, Vingmed, Horten, Norway) using a 2.5-MHz probe for image acquisition. The images were acquired in parasternal long and short axis as well as in apical four- and two-chamber projections. Left atrial and LV dimensions and function were assessed by standard methods and LV volumes and ejection fraction were calculated by the Simpson's method, averaging values from three consecutive cardiac cycles[30].

### Tissue Doppler Echocardiography (TDE)

Images were obtained from the apical four and two-chamber views as well as from the apical long-axis view. The pulse repetition frequency and the ultrasound sector beam image width and depth were modulated to avoid the Nyqvist's upper frequency while obtaining images with at least 100 frames per second. Five consecutive cardiac cycles were acquired during post-expiratory apnea in each of the above-mentioned projections and the images were analyzed off-line using an EchoPAC™ 6.3.6 software (General Electric, Horten, Norway).

**Figure 1:**
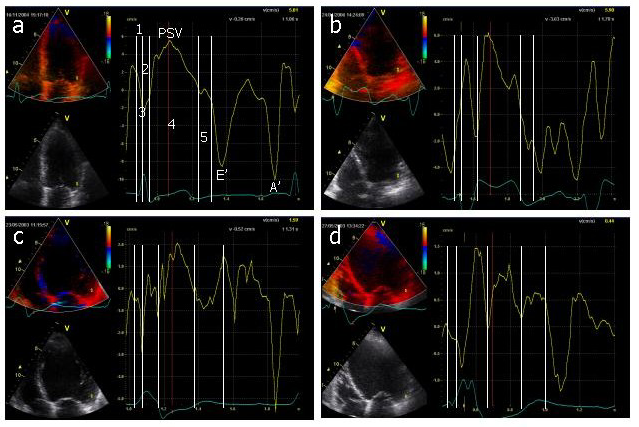
**a. Echocardiographic image showing the apical four-chamber view in a subject of the control group.** The image shows the myocardial velocity profile obtained in the basal segment of the left ventricular lateral wall. The time intervals identified by figures indicate: 1 = Electromechanical delay time (EMDT); 2 = Isovolumic contraction time (IVCT); 3 = Electro hemodynamic delay time (EHDT); 4 = Ejection time (ET); 5 = Isovolumic relaxation time (IVRT). A' = Late diastolic (A-wave) velocity); E' = Early (E-wave) diastolic velocity; PSV = Peak systolic velocity. *b*, represent the same image in a patient with isolated LBBB; *c *in a patient with dilated cardiomyopathy and LBBB; *d *in a patient with ischemic cardiomyopathy and LBBB.

A 2 mm sample volume was placed at the basal segment of the LV near the A-V plane in the following walls: the posterior septum and lateral walls (in the 4-chamber view), inferior and anterior walls (in the two-chamber view), and posterior wall and anterior septum (in the long-axis apical view). As previously described[29] and as shown in figure 1a, MC was studied from the myocardial velocity profile curves, calculating the following temporal events during the cardiac cycle: The *electro-mechanical delay time*, the *electro-hemodynamic delay time*, the *isovolumic contraction time*, the *ejection time *and the *isovolumic relaxation time*. The *electro-mechanical delay time *was defined as the time interval between the start of the QRS complex and the beginning of the mechanical activity on the myocardial velocity curve. The *electro-hemodynamic delay time *was defined as the time interval between the start of the QRS complex and the beginning of the ejection time. The *isovolumic contraction time *was calculated by subtracting the electro-mechanical delay time from the electro-hemodynamic delay time. The *ejection time *was defined as the time interval between the opening and closure of the aortic valve characterized by the descent of the myocardial velocity profile curve to the baseline. The *isovolumic relaxation time *was defined as the time interval between the closure of the aortic valve and the opening of the mitral valve characterized by the beginning of the E' wave; when a post-systolic velocity was present, the beginning of the isovolumic relaxation time was measured at the peak of that velocity. Using TDE, the LV systolic function was calculated by means of the peak systolic velocity (PSV) of all LV walls and by the temporal integration of the PSV curve, a measure of the A-V plane displacement that assess the longitudinal LV systolic function. All the above-mentioned measures are expressed as the average of the values obtained at each of the basal segments of the six LV walls.

### Statistical analysis

The values are expressed as means ± standard deviation. ANOVA was used to test differences among groups, followed by the Tukey test for post-hoc comparisons between groups. The correlation coefficient was used to establish relationships between variables expressing the LVSF and variables expressing MC. A *P *value < 0.05 was considered statistically significant.

## Results

As shown in table 1, the Controls were older than the other groups (P < 0.001 for all comparisons). Age was however similar among patients with isolated LBBB and patients with DCMP and with ICMP. Males and females were similarly represented in all groups, except in patients with DCMP and with ICMP in which males were over represented. Differences observed in left atrial diameter, septum and posterior wall thicknesses, LV end-diastolic diameter and volumes, and LVEF are shown in table 1. No differences were found between DCMP and ICMP groups regarding those variables; however, significantly differences were found between DCMP and ICMP groups compared with Controls and compared with the LBBB group.

**Table 1:** Demographics and standard echocardiographic variables.

**Variables**	**Controls N = 20**	**LBBB N = 21**	**ICMP N = 19**	**DCMP N = 26**	**P**
Age, years	70 ± 4	61 ± 8*	61 ± 9*	65 ± 9*	< 0.001
Gender, (M/F)	10/10	11/10	17/2	21/5	0.01
QRS, ms	84 ± 4	145 ± 9*	150 ± 9*	154 ± 9*	< 0.001
LA diameter, mm	34 ± 5	37 ± 8	49 ± 10*†	48 ± 10*†	<0.001
SWT, mm	11 ± 0.7	11 ± 1.3	9.7 ± 1.9†	9.2 ± 1.9*†	< 0.01
PWT, mm	11 ± 1.0	10 ± 1.0	10 ± 2.1	10 ± 1.8	NS
LVEDD, mm	43 ± 6	46 ± 6	75 ± 8*†	72 ± 12*†	<0.001
LVEDV, ml	99 ± 23	114 ± 36	247 ± 73*†	274 ± 123*†	< 0.001
LVESV, ml	46 ± 10	63 ± 26	177 ± 62*†	203 ± 105*†	< 0.001
LVEF, %	54 ± 4	46 ± 8*	27 ± 10*†	29 ± 7*†	<0.001

**Abbreviations**: LA = Left atrium; LVEDD = Left ventricular end diastolic diameter; LVEF = Left ventricular ejection fraction; LVEDV = Left ventricular end diastolic volume; LVESV = Left ventricular end systolic volume; PWT = Posterior wall thickness; SWT = Septal wall thickness.

P = Analysis of variance.

* < 0.05 vs. Controls.

†< 0.05 vs. LBBB.

Table 2 shows all measures of MC assessed by TDE in the entire population. The *electro-mechanical delay time *was shorter in the Controls group than in the other groups (P < 0.001 for all comparisons); however, this interval was similar among LBBB, DCMP and ICMP groups. The *electro-hemodynamic delay time *was shorter in the Controls group than in the other groups (P < 0.001 for all comparison); it was also shorter in the DCMP and ICMP groups as compared with the LBBB group (P < 0.01 for both comparisons), and shorter in the ICMP group than in the DCMP group (P < 0.01). Findings similar to these were observed with respect to the *isovolumic relaxation time*. The *isovolumic contraction time *was shorter in the Controls group than in the other groups (P < 0.001 for all comparisons); it was shorter in the ICMP and the DCMP groups as compared with the LBBB group (P < 0.01 for both comparisons), but was similar in the DCMP and the ICMP groups. Findings similar to these were observed with respect to the *ejection time*. The PSV was higher in the Controls group as compared with the other groups (P < 0.001 for all comparisons); it was lower in the DCMP and ICMP groups as compared with the LBBB group (P < 0.01 for both comparisons), but it was similar in the DCMP and ICMP groups. Findings similar to these were observed for the A-V plane displacement variable.

**Table 2:** Parameters of myocardial coordination and left ventricular systolic function obtained by tissue Doppler echocardiography.

**Variables**	**Controls N = 20**	**LBBB N = 21**	**ICMP N = 19**	**DCMP N = 26**	**P**
EMDT, ms	14 ± 1	45 ± 4*	42 ± 9*	47 ± 12*	< 0.001
EHDT, ms	81 ± 10	136 ± 10*	136 ± 25*	163 ± 33*†‡	< 0.001
IVCT, ms	67 ± 9	91 ± 8*	96 ± 18*	116 ± 31*†	< 0.001
IVRT, ms	70 ± 10	134 ± 18*	127 ± 34*†	160 ± 29*†‡	<0.001
ET, ms	321 ± 20	278 ± 41*	207 ± 28*†	220 ± 44*†	< 0.001
PSV, cm/s	6.3 ± 1.1	4.1 ± 0.8*	2.6 ± 0.8*†	3.2 ± 1.0*†	< 0.001
A-V, mm	9.7 ± 1.5	8.0 ± 1.8*	3.9 ± 1.6*†	5.6 ± 2.8*†	<0.001

**Abbreviations**: A-V = Atrio-ventricular plane displacement; EHDT = Electro-hemodynamic delay time; EMDT = Electro-mechanical delay time; ET = Ejection time; IVCT = Isovolumic contraction time; IVRT = Isovolumic relaxation time; PSV = Peak systolic velocity.

P = Analysis of variance.

* < 0.05 vs. Controls.

†< 0.05 vs. LBBB.

‡ < 0.05 vs. ICMP.

**Figure 2:**
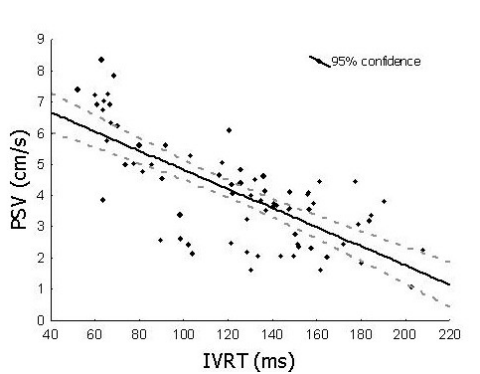
Scatter plot of the correlation coefficient (r = -0.74) between a variable expressing the left ventricular systolic function, (PSV = peak systolic velocity) and a variable expressing the left ventricular myocardial coordination (IVRT = the isovolumic relaxation time).

Table 3 shows the correlation coefficients between several variables of LV systolic function and MC. Of interest is to note the very good correlation coefficient between the *isovolumic relaxation time *and the PSV (see figure 2), between the *electro-hemodynamic delay time *and the PSV, and between the *ejection time *and the A-V plane displacement. The correlation coefficient between the other studied variables was in between fair and good.

**Table 3:** Correlation coefficients corresponding to variables expressing myocardial coordination and variables expressing left ventricular systolic function.

	**EMDT**	**EHDT**	**IVCT**	**IVRT**	**ET**
**LVEF**	-0.53	-0.63	-0.59	-0.63	0.64
**PSV**	-0.61	-0.71	-0.63	-0.74	0.68
**A-V**	-0.45	-0.53	-0.49	-0.59	0.74

**Abbreviations**: A-V = Atrio-ventricular plane displacement; EHDT = Electro hemodynamic delay time; EMDT = Electro mechanical delay time; ET = Ejection time; IVCT = Isovolumic contraction time; IVRT = Isovolumic relaxation time; LVEF = Left ventricular ejection fraction; PSV = Peak systolic velocity.

## Discussion

The present study showed that: 1) LBBB considerably alters MC as evidenced by a prolongation of the electro-mechanical delay time, the electro-hemodynamic delay time, the isovolumic contraction time and the isovolumic relaxation time, and by a shortening of the ejection time; 2) In patients with DCMP and ICMP these derangements were more pronounced; 3) Some variables, viz. the electro-hemodynamic delay time and the isovolumic relaxation time made it possible to differentiate patients from the DCMP and the ICMP groups; 4) A good or very good coefficient of correlation was found between variables of MC and of LV systolic function demonstrating that the larger the derangement in MC, the poorer the LV systolic function. The present findings may explain the mechanisms of poorer LV systolic function and the poorer prognosis in patients with cardiomyopathies with LBBB than in those without LBBB [9-13].

As known, the myocardial coupling and uncoupling processes (electro-hemodynamic delay time and the isovolumic relaxation time) are energy demanding[31], indicating that their excessive prolongation imply a higher energy expenditure with a less effective LV hemodynamic work. In the present study, an exaggerated prolongation of the electro-mechanical and electro-hemodynamic delay times resulted in a remarkable shortening of the ejection time that shortened the effective LV hemodynamic working time. On the other hand (although not shown in the present study) a striking prolongation of the isovolumic relaxation time could significantly shorten the LV early filling time, a fact that in presence of A-V conduction disturbances (a common finding in patients with cardiomyopathies) could lead to a rise in the LV end-diastolic pressure, to an impairment of the LV filling during atrial systole, and to a generation of diastolic mitral regurgitation that finally, could contribute to an additional diminution of the LV stroke volume.

The good correlation coefficient between the variables of MC and LV systolic function does not necessarily establish a cause-effect relationship between them, though it is probable that the poor or the lack of MC found in patients with LBBB and cardiomyopathies could be an additional cause of deterioration of LV systolic function seen in these patients. The intrinsic poor contractile function together with the hemodynamic imbalance induced by the inter- and intra-ventricular dyssynchrony usually observed in these patients are additional known factors for poor LV systolic function. To what extent the lack of MC or the inter- and intra-ventricular dyssynchrony contribute to the deterioration of the LV systolic function and to what extent these processes coexist is difficult to ascertain. Surely all these mechanisms (poor contractility, lack of MC and inter- and intra-ventricular dyssynchrony) alone or together play an important role in the deterioration of LV systolic function.

Though some parameters of MC were more altered in patients with DCMP than in patients with ICMP, the current results cannot yet be used to make the differential diagnosis of these entities. The findings may, however, lead to future investigations for testing new hypothesis; for example, although the coupling and uncoupling processes were more prolonged in patients with DCMP than in those with ICMP, the LV systolic function was poorer in the last group as compared with the former (lower values of PSV and A-V plane displacement). This apparent contradiction may be explained by the fact that patients with DCMP may have a myocardial contractile reserve that is larger than in patients with ICMP. It could then be hypothesized that patients with moderately depressed myocardial contractile reserve but severely depressed MC (patients with DCMP) may better respond to CRT than those with severely depressed myocardial contractile reserve but with only moderately depressed MC (patients with ICMP). Indeed, the subgroup analysis of some cohort studies[25,32] and of a randomized trial[18] of patients treated with CRT points into that direction. In addition one of the independent predictive factors of clinical outcome in patients with CRT was a preserved myocardial contractile reserve assessed during dobutamine stress echocardiography[33].

Although TDE has not been used to select patients for CRT in large randomized clinical trials, this technique together with other methods have been used to estimate the results of such therapy[26,34-37]. However, most of the publications refer to the evaluation of the changes in the LV systolic function after CRT and practically none of the mentioned studies addressed the evaluation of the effects of CRT on MC. In addition, the number of non-responders to CRT in some of the above mentioned studies is much larger when the response is evaluated in terms of improvement of echocardiographic variables of reverse remodeling[22,24,26] than when the response is evaluated using subjective or relatively objective parameters of functional capacity [23,38-41], which may in some way question the validity or the usefulness of the echocardiographic variables studied a fact that has recently been tested in the PROSPECT trial[42]. One possible explanation for these results rests on the fact that the time to peak systolic velocity or the time to peak systolic strain or strain rate used in most of the mentioned studies[24,26,43,44] are parameters extremely difficult to reliably assess in patients with severely reduced LV systolic function; as seen figure 1c and 1d, the time to PSV is difficult to determine accurately. Assessing the other different time intervals by TDE technique as done in the present study may represent an alternative way to assess both MC and cardiac dyssynchrony that may help to a better selection of candidates for CRT as shown in the PROSPECT trial in which the left ventricular preejection interval and time to onset of systolic velocity (parameters expressing MC) were the only echocardiographic parameter that predicted response to CRT[42].

### Study limitations

As the main aim of the present study was to evaluate the effects of LBBB on MC and of it's repercussion on LV systolic function, the study did not include patients with cardiomyopathies without LBBB; therefore we could not assess the degree of MC and it's effect on LVSF in that group of patients, as shown in previous studies [45,46]. Although the temporal events during the cardiac cycle have been studied with some reliability and confidence by means of TDE in normal individuals [8], the reproducibility of those temporal events needs to be confirmed in patients with DCMP or with ICMP by other investigators. Although all parameters of MC were obtained by averaging the values obtained in the six basal LV segments, it has to be acknowledged that those values, due to the reasons intrinsically related to the acquisition technique, were not simultaneously obtained during the same cardiac cycle.

## Conclusion

LBBB introduces severe derangements in the process of MC that are more pronounced in patients with cardiomyopathies. There are good correlations between the variables expressing MC and LV systolic function, indicating that these processes are closely related irrespective of background pathologies associated with LBBB, and that one important determinant of deterioration of LV systolic function could be the degree of loss of MC. The electrophysiological, electromechanical, and hydraulic events that represent MC may help to differentiate patients with DCMP from those with ICMP and that may be used as additional criteria for patient selection for CRT.

## List of abbreviations

A-V: Atrio-Ventricular; DCMP: Dilated cardiomyopathy of unknown origin; EHDT: Electro-hemodynamic delay time; EMDT: Electro-mechanical delay time; ET: Ejection time; ICMP: Ischemic cardiomyopathy of ischemic origin; IVCT: Isovolumic contraction time; IVRT: Isovolumic relaxation time; LBBB: Left bundle branch block; LV: Left ventricle (ventricular); MC: Myocardial coordination; PSV: Peak systolic velocity; TDE: Tissue Doppler Echocardiography.

## Authors' contributions

MQ: Made substantial contributions to conception and design, or acquisition of data, or analysis and interpretation of data; mainly responisble for drafting the manuscript and gave final approval of the version to be published. SS: Contributed with data acquisition and interpretation, participated in drafting process and approved the final version. SG: Contributed with data acquisition and interpretation, statistical analysis, participated in drafting process and approved the final version. LÅB: Made substantial contributions to conception and design, participated in drafting process and approved the final version. FdF: Contributed with data acquisition and interpretation, statistical analysis, participated in drafting process and approved the final version. VB: Made substantial contributions to conception and design, participated in drafting process and approved the final version.
